# A Biomass‐Based Integral Approach Enables Li‐S Full Pouch Cells with Exceptional Power Density and Energy Density

**DOI:** 10.1002/advs.202101182

**Published:** 2021-05-24

**Authors:** Yuping Liu, Yvo Barnscheidt, Manhua Peng, Frederik Bettels, Taoran Li, Tao He, Fei Ding, Lin Zhang

**Affiliations:** ^1^ Institute of Solid State Physics Leibniz University Hannover Appelstrasse 2 Hannover 30167 Germany; ^2^ Laboratory of Nano and Quantum Engineering (LNQE) Leibniz University Hannover Schneiderberg 39 Hannover 30167 Germany; ^3^ Institute of Electronic Materials and Devices Leibniz University Hannover Schneiderberg 32 Hannover 30167 Germany

**Keywords:** biomass‐based porous carbon matrices, dendrite‐free Li anodes, electric vehicles/grid storage, Li‐S pouch cells, superior energy/power densities

## Abstract

Lithium‐sulfur (Li‐S) batteries, as part of the post‐lithium‐ion batteries (post‐LIBs), are expected to deliver significantly higher energy densities. Their power densities, however, are today considerably worse than that of the LIBs, limiting the Li‐S batteries to very few specific applications that need low power and long working time. With the rapid development of single cell components (cathode, anode, or electrolyte) in the last few years, it is expected that an integrated approach can maximize the power density without compromising the energy density in a Li‐S full cell. Here, this goal is achieved by using a novel biomass porous carbon matrix (PCM) in the anode, as well as N‐Co_9_S_8_ nanoparticles and carbon nanotubes (CNTs) in the cathode. The authors' approach unlocks the potential of the electrodes and enables the Li‐S full pouch cells with unprecedented power densities and energy densities (325 Wh kg^−1^ and 1412 W kg^−1^, respectively). This work addresses the problem of low power densities in the current Li‐S technology, thus making the Li‐S batteries a strong candidate in more application scenarios.

## Graphical abstract

The Li‐S full pouch cells with unprecedented power densities and energy densities are achieved using a biomass‐enabled integral approach: a novel biomass porous carbon matrix in the anode, as well as N‐Co_9_S_8_ nanoparticles and carbon nanotubes in the cathode, unlocks the potential of the electrodes, thus making the Li‐S batteries a strong candidate in more application scenarios.

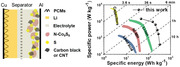

## 1 Introduction

To meet the rise of electric vehicles and grid/home energy storage, rechargeable batteries with superior energy density (Wh kg^−1^), as well as power density (W kg^−1^), are urgently required.^[^ ^1^, ^2^ ^]^ For the dominant LIBs, their energy density and power density are reaching the practical limits and can hardly go beyond 300 and 1000 W kg^−1^, respectively.^[^ ^2^, ^3^, ^4^ ^]^

Among the promising new chemistries, anion‐redox Li‐S batteries are widely seen as a top candidate because of their high theoretical cathode energy density of 2600 Wh kg^−1^ based on the weight of S_8_.^[^ ^5^, ^6^ ^]^ However, the development of practical Li‐S full cells is still hampered by a number of challenges. For the anode, the Li dendrite growth leads to low Coulombic efficiency (CE), shortened cycle life, and even serious safety concerns.^[^ ^7^, ^8^ ^]^ For the cathode, the dissolution of lithium polysulfides (LiPSs) intermediates in the electrolyte (so‐called “shuttling effect”) and the sluggish redox kinetics of S_8_ ↔ Li_2_S will cause dramatic capacity fading and low CE, especially with high sulfur mass loadings, and cycled under large current densities.^[^ ^9^ ^]^

To solve these issues, a considerable amount of research has been devoted to improving the individual cell components of Li‐S batteries. For example, advanced electrodes with strong anchoring for LiPSs and fast LiPSs conversion have been demonstrated.^[^ ^10^ ^]^ The fast plating/striping for Li anode has also been realized.^[^ ^11^ ^]^ For practical applications, electrode designs that can accommodate high sulfur mass loading, low (electrolyte/sulfur) *E/S* ratio, and large current density have been reported. Thanks to these achievements, it can be expected that integrated approaches will be developed for high‐performance Li‐S full cells, by considering a favorable mix of the performance parameters of each component.^[^ ^12^, ^13^ ^]^

There have been a few attempts along this direction, but only with relatively low active material mass loadings.^[^ ^13^, ^14^ ^]^ Moreover, today's Li‐S batteries have low power densities, limiting their applications to very few specific scenarios that need low power, low mass, and long working time (for example, high altitude long endurance unmanned aerial vehicles – HALE UAVs). In fact, most of the Li‐S studies today focus on improving the energy density, but overlook the importance of power density. This is probably due to the fact that many of the electrode protection strategies, which are used to enhance the energy density, tend to passivate the electrodes and cause a slow ionic kinetics and thus reduce the power density.

However, power density is important in a much wider range of application scenarios, for example, in contemporary electric vehicles (especially, during acceleration and uphill driving) and in grid/home energy storage. Interestingly, in the so‐called “Ragone plots”,^[^ ^15^ ^]^ Li‐S batteries are expected to show higher power densities than that of LIBs, which is unfortunately not the case in real situations. This problem becomes even more severe when using more realistic parameters, such as high sulfur mass loadings (>5 mg cm^−2^) and low *E/S* ratio (<2 µL mg^−1^), etc.,^[^ ^16^ ^]^ in the Li‐S pouch cells. Therefore, it is highly demanded to develop practical Li‐S full (pouch) cells with superior energy density (>300 Wh kg^−1^) and power density (>1000 W kg^−1^) at the same time. Furthermore, the pouch cells are more fragile than the coin cells, and they are easier to fail during cycling. The recent researches on the failure mechanisms in pouch cells reveal that the limited diffusion kinetics causes uneven electrochemical reactions in both the Li anode and sulfur cathode, together with the electrolyte depletions across the electrode and the thermal runaway. This leads to the failure of the pouch cells, especially those with high S mass loadings.^[^ ^17^, ^18^ ^]^

Herein, this work shows that the ambitious goals outlined above can be achieved via the following three steps: 1) a novel biomass‐based PCM has been developed and used as the Li metal host in the anode, which can homogenize the flux of Li ions and inhibit the Li dendrite growth. This leads to a significant improvement of the full cell performances, especially at large areal capacities and high current densities; 2) N‐Co_9_S_8_ nanoparticles used in the cathode can efficiently immobilize and catalyze the LPSs during the conversion, which is another contributing factor for the accelerated reaction kinetics and the improved power density;^[^ ^19^ ^]^ 3) the cathode was constructed by mixing S, N‐Co_9_S_8_ with CNTs, where the CNTs can simultaneously boost the electrical and mechanical properties of the cathode. This is particularly important at high sulfur mass loadings and low *E/S* ratios, and is crucial for a high S utilization and thus high specific capacity (energy density).^[^ ^20^ ^]^ This integrated approach enables the Li‐S full pouch cells with superior energy and power densities (325 Wh kg^−1^ and 1412 W kg^−1^, respectively), which are considerably higher than that of the state‐of‐the‐art LIBs. To the best of our knowledge, this is the first demonstration of superior energy/power densities simultaneously in a Li‐S pouch cell, with high S mass loadings and using low *E/S* ratios.

## 2 Results and Discussion

### 2.1 The Preparation and Characterization of PCMs

**Figure 1:**
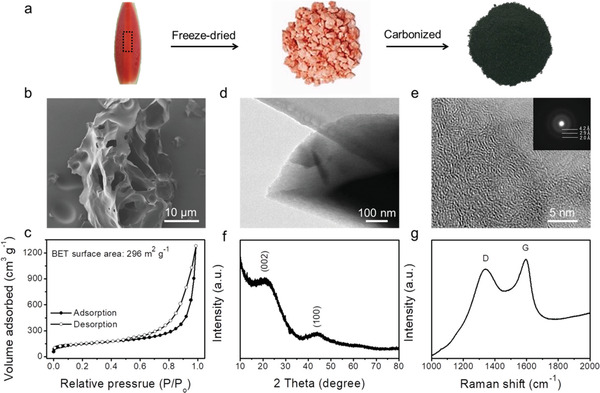
The morphology and porous structure of the PCMs. a) Schematic illustration of the fabrication process, b) SEM image, c) nitrogen adsorption–desorption isotherm, d) TEM image, e) HRTEM image, f) XRD patterns, g) Raman spectra of the PCMs. The insert of (e) is the SAED image.

The PCMs have been synthesized by using the watermelon flesh as a source of carbon. The watermelon flesh has a porous structure with a high surface area. Hence it has a great potential to be used as a 3D host material, for a uniform Li melt infiltration/plating and fast electron/ion transportation. To maintain its porous structure, the watermelon flesh was first treated with a freeze‐dried process, and followed by carbonization, see **Figure** **1a**. The detailed process can be found in the experimental section. This novel concept provides a scalable and versatile method for producing highly porous carbon matrices.

The morphology of the PCMs was investigated by scanning electron microscopy (SEM). The SEM images prove the honeycomb‐like porous structure and the large surface area (Figure 1b and Figure S1, Supporting Information), indicating that the unique honeycomb‐like network of the watermelon flesh was preserved after the freeze‐drying and carbonization. The N_2_ adsorption–desorption isotherms revealed a large BET surface area of 296 m^2^ g^−1^ (Figure 1c). This, together with the excellent conductivity, will facilitate a fast plating of Li at large areal capacities. The morphology of the PCMs was also characterized by transmission electron microscopy (TEM) (Figure 1d,e). The HRTEM suggests an amorphous structure of carbonized watermelon flesh, and the selected area electron diffraction (SAED) (inset of Figure 1e) shows a few bright ring patterns corresponding to the *d‐*spacing of 4.2, 2.9, and 2.0 Å, respectively.

The X‐ray diffraction (XRD) pattern and Raman spectra were measured to further determine the components of the as‐prepared PCMs. As shown in Figure 1f, two broad diffraction peaks at around 22° and 44° were observed, which correspond to the (002) and (100) planes of the graphitic carbon, respectively.^[^ ^21^ ^]^ The Raman spectra further elaborate the structures of the as‐prepared carbon material, revealing two pronounced peaks—D band at about 1338 cm^−1^ from the disordered *sp^3^*‐C atoms and G band at about 1595 cm^−1^ from the in‐plane bond‐stretching motion of pairs of *sp^2^*‐C atoms, respectively.^[^ ^22^ ^]^ The intensity of the G band is higher than that of the D band, indicating a higher in‐plane bond‐stretching motion of *sp^2^*‐C atoms (graphitization) in the PCMs.

The nanostructured carbon was first used as the sulfur host by Nazar's group,^[^ ^5^ ^]^ then varieties of bio‐derived materials have been developed in Li‐S batteries to suppress the shuttle effect over the past few years.^[^ ^23^ ^]^ However, recent studies show that the nonpolar carbon‐based sulfur hosts can only achieve limited improvement in Li‐S batteries, due to their weak interaction with the polar LiPSs.^[^ ^24^ ^]^ On the other hand, the porous carbons can regulate the nucleation process of metallic Li electrodeposition as well as the subsequent growth process, as shown in the milestone work from Cui's group.^[^ ^7^ ^]^ Thus, the research interest of the bio‐derived materials is moving from the sulfur host to the Li metal host.

The Li@PCMs anode was prepared by a melt infusion process (see method). Benefiting from the large surface area and the capillary forces generated by PCMs, the lithium melt can be easily infused into the pore channels when a PCMs sheet is placed on the surface of the molten Li (Figure S2b, Supporting Information). Finally, the composite anode was immersed into the electrolyte to form a stable solid electrolyte interphase (SEI) layer (Figure S2d, Supporting Information). Due to the low density and high porosity of PCMs, Li@PCMs was able to achieve a high specific capacity of 3487 mAh g^−1^ after being charged to 1.0 V versus Li/Li^+^ under 100 µA cm^−2^ (specific capacity calculated by the weight of the Li metal, Figure S3, Supporting Information), which is > 90% of the theoretical specific capacity of Li metal (3861 mAh g^−1^). The newly developed PCMs provide a much higher specific capacity when compared to most of the carbon‐based Li metal host.^[^ ^25^ ^]^ This indicates that the PCMs host does not diminish the advantage of the high capacity of Li metal. We also observed that the electrode completely turns black after the charging (Figure S2c, Supporting Information).

### 2.2 Li Metal Electrochemical Plating/Striping

**Figure 2:**
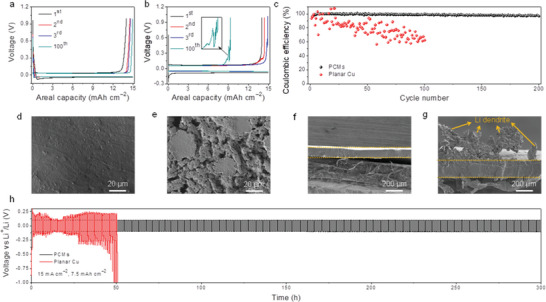
The electrochemical plating/striping of the PCMs host and the planar Cu. Voltage profiles of the Li plating/stripping: a) PCMs and b) planar Cu, insert is the enlarged curve. c) Comparing CE of the PCMs (black) and the planar Cu (red). Top‐view SEM images of the anodes after plating: d) PCMs and e) planar Cu. Cross‐sectional SEM images of the anodes after plating: f) PCMs and g) planar Cu. (d–g) were measured with an areal capacity of 15 mAh cm^−2^ and under a current density of 3 mA cm^−2^. h) Symmetric cell cycling performances: Li@PCMs (black) and Li@Cu (red), with an areal capacity of 15 mAh cm^−2^ under a current density of 7.5 mA cm^−2^.

Li plating/stripping experiments were then performed, in order to investigate the nucleation overpotential and the CE of Li metal anodes, see **Figure** **2**. The Li nucleation was studied by the nucleation barrier at the initial stage of Li deposition. With a large areal capacity (15 mAh cm^−2^) and a high current density (3 mA cm^−2^), the PCMs host shows a lower plating overpotential of ≈ 30 mV compared to ≈ 50 mV of planar Cu (Figure S4, Supporting Information). After the initial cycle, the planar Cu shows severe fluctuations during striping, while the PCMs host demonstrates a much stable striping process. And even after 100 cycles, the PCMs host still shows a relatively low plating/striping overpotential, and no obvious increase can be observed. Figure 2c shows the average CE (98.79%) of PCMs after 200 cycles under a high current density with a large Li capacity, which is much higher than that of the planar Cu (62.32% after 100 cycles). Furthermore, even with a much larger areal capacity (30 mAh cm^−2^), the PCMs host still displays stable voltage profiles and a relatively high average CE (98.46% after 50 cycles, Figure S5, Supporting Information). In addition, the morphology of anodes after plating was measured by SEM. As shown in Figure 2d, the Li@PCMs showed an extremely flat surface without any obvious crevices, confirming the outstanding reversibility of the dense nucleation on the PCMs and the high utilization of the PCMs for Li deposition. In comparison, Li was sparsely deposited on the planar Cu host, forming a mossy‐like loose texture with large crevices, which can be attributed to the growth of Li dendrites (Figure 2e). These can be further confirmed by the cross‐sectional SEM images in Figure 2f,g.

Furthermore, symmetric cells with a large Li deposition capacity (15 mAh cm^−2^) were cycled under a high current density (7.5 mA cm^−2^), in order to evaluate the interfacial stability of Li metal anodes, see Figure 2h. The Li@Cu symmetric cell exhibits random voltage oscillations and a significantly shorter cycle life, which can be attributed to the Li dendrite growth and the unstable interfaces during the repeated plating/striping. In contrast, the Li@PCMs symmetric cell demonstrates a stable voltage profile with a low polarization (only ≈ 100 mV) and an ultralong life (> 300 h) (Figure 2h and Figure S6, Supporting Information). This is better than the previously reported results in terms of high current density, large Li capacity, and low polarization.^[^ ^13^, ^26^ ^]^

Importantly, recent studies reveal that the pouch cell failure may be mainly due to the Li anode degradation because of the “deep” cycling of Li metal, especially with a high S loading.^[^ ^17^, ^27^ ^]^ So far, most of the works on Li‐S batteries focused mainly on the sulfur cathode, but didn't recognize the importance of the Li anode. This work provides useful insights that the full potential of Li‐S full pouch cells can be unlocked by optimizing both the Li anode and sulfur cathode.

### 2.3 Electrochemical Performance of the N‐Co_9_S_8_/S Electrode

**Figure 3:**
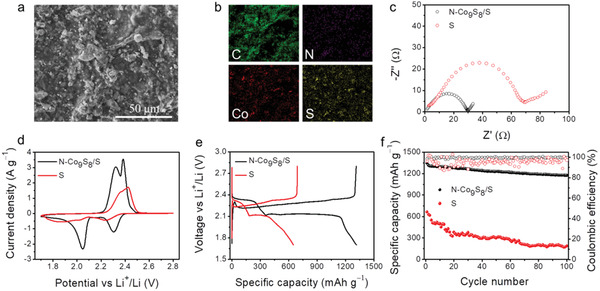
The electrochemical performance of the N‐Co_9_S_8_/S electrode compared with that of the S electrode. a) SEM, b) EDS mapping of C, N, Co, and S, respectively, c) EIS spectra, d) CV curves at the scan rate of 0.1 mV s^−1^, e) charge/discharge profiles at the current density of 0.2 A g^−1^, and f) cycling performances.

As discussed above, the full capability of a Li‐S full cell depends on the synergism between the Li anode and the sulfur cathode. Considering the dissolution of LiPSs in the electrolyte and the sluggish redox kinetics of S_8_ ↔ Li_2_S, we used the N‐Co_9_S_8_ nanoparticles mixed with sulfur (N‐Co_9_S_8_/S) as the cathode. This is because the N‐Co_9_S_8_ nanoparticles can effectively immobilize and catalyze the LiPSs during its conversion, which has been demonstrated in our recent work.^[^ ^19^ ^]^ The structural and morphological characterizations of N‐Co_9_S_8_ nanoparticles are shown in Figure S7, Supporting Information. The morphology of the N‐Co_9_S_8_/S composite is shown in **Figure** **3a**, and the corresponding element mappings of C, N, Co, and S reveal the uniform chemical compositions (Figure 3b).

The electrochemical performances for the obtained N‐Co_9_S_8_/S_8_ composites were evaluated in half‐cells (paired with Li foil as the counter and reference electrode). The electrochemical impedance spectroscopy (EIS) of the N‐Co_9_S_8_/S electrode shows a much smaller semicircle compared to that of the S electrode, indicating a faster charge transfer behavior and improved kinetics (Figure 3c). The cyclic voltammetry (CV) curve of the N‐Co_9_S_8_‐based electrode shows two pairs of redox peaks (O_1_, O_2_, R_1_, and R_2_, scan rate: 0.1 mV s^−1^, Figure 3d). Importantly, a considerably mitigated electrochemical polarization and increased peak current density can be achieved by the N‐Co_9_S_8_/S electrode, especially for the transformation from the higher‐order LiPSs to the low order LiPSs (about 2.1 V). This demonstrates the faster kinetic and higher S utilization of the N‐Co_9_S_8_/S electrode (Figure S8, Supporting Information). And this is further supported by the galvanostatic charge/discharge test (0.2 A g^−1^, Figure 3e, and Figure S9, Supporting Information), where the overpotential is 200 mV for the S electrode and only 100 mV for the N‐Co_9_S_8_/S electrode, and the specific capacity of the S electrode is about 50% to that of the N‐Co_9_S_8_/S electrode. The cycling performances are shown in Figure 3f. The N‐Co_9_S_8_/S electrode shows a capacity retention of 1174 mAh g^−1^ and an average CE of 99.38% over 100 cycles, both of which are much higher than those of the S electrode (186 mAh g^−1^ and 95.25%, respectively). The superior cyclability of the N‐Co_9_S_8_/S electrode has been discussed in detail in our recent work.^[^ ^19^ ^]^

### 2.4 Electrochemical Performance of the Li@PCMs||N‐Co_9_S_8_/S Full Coin Cell

**Figure 4:**
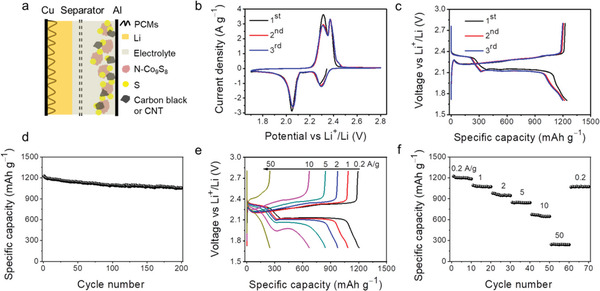
The electrochemical performance of the Li@PCMs||N‐Co_9_S_8_/S full coin cell. a) Schematic illustration of the structure of Li−S full cells, b) CV profiles (scan rate: 0.1 mV s^−1^), c) charge/discharge curves (current density: 0.2 A g^−1^), d) long‐term cycling performance (current density: 0.2 A g^−1^), e) voltage profiles at different current densities, and f) rate performance. (S mass loading: 1 mg cm^−2^, the areal capacity of Li composite: 2 × excess of S (3.35 mAh cm^−2^), and the thickness of Li composite is about 30 µm.)

Li‐S full cells were then constructed to take advantage of the superior performances of both Li@PCMs anode and N‐Co_9_S_8_/S cathode. **Figure** **4a** represents the schematic diagram of the Li‐S coin cell (with 2 × excess Li@PCMs anode). The CV curves of the Li‐S full cell show two couples of reversible reduction (located at 2.3 and 2.06 V) and oxidation peaks (located at 2.31 and 2.38 V), as shown in Figure 4b. The peak at 2.3 V corresponds to the polymerization of sulfur into long‐chain LiPSs (Li_2_S*_n_*, 4 < *n* < 8), and the reduction peak at 2.1 V indicates the further reduction of the long‐chain LiPSs to the short‐chain LiPSs (Li_2_S*_n_*, *n* ≤ 2). The two oxidation peaks at 2.3 and 2.4 V are associated with the reverse transformation from the short‐chain LiPSs to the long‐chain LiPSs and finally to the S_8_, respectively. We note that the redox peaks almost overlap for the 2nd and 3rd cycles, which is the indication of excellent reversibility for the electrochemical processes. The typical charge/discharge profiles are shown in Figure 4c, which are similar to the results from the half cell. The Li@PCMs||N‐Co_9_S_8_/S full cell is also investigated by the long term galvanostatic cycling test. Due to the highly efficient utilization of both anode and cathode, the full cell exhibits excellent cycling stability with a high capacity retention of 1054 mAh g^−1^ over 200 cycles (Figure 4d).

The large plating/striping capacity/current density of Li@PCMs anode, as well as the unique catalytic function of N‐Co_9_S_8_, can accelerate the kinetic conversion of S_8_ ↔ Li_2_S to improve the rate performance of the Li‐S full cell. As shown in Figure 4e,f, the full cell could still maintain a high reversible capacity of 240 mAh g^−1^ even at an ultrahigh current density (50 A g^−1^). The specific capacity restores to 1067 mAh g^−1^ when the current returns from 50 to 0.2 A g^−1^. It is worth noting, that the charge/discharge profiles show two well‐defined plateaus at about 2.1 V and low overpotentials even at high current densities, strongly proving the excellent reaction kinetics. In contrast, without the N‐Co_9_S_8_ additive and the PCMs host, severe shuttling behavior, large overpotential, and dramatic capacity decay were observed for the Li@Cu||S full cell (Figure S10, Supporting Information).

Despite the high theoretical energy density, however, it is very challenging to achieve high energy density and high power density simultaneously in a practical Li‐S full cell. There are stringent requirements on the sulfur mass loading (≥ 5 mg cm^−2^), Li areal capacity deposition (≥ 10 mA cm^−2^), current density (≥ 10 A g^−1^), and the *E*/*S* ratio. This imposes a great challenge when constructing the cathode, especially for a pouch cell. The traditional carbon black shows limited electrical and mechanical properties in the high S mass loading cathode (compared with the CNTs), thus cannot be used to maximize the specific capacity of the electrode without sacrificing the power density (Figure S11, Supporting Information).

### 2.5 Power and Energy Densities of the Li@PCMs||N‐Co_9_S_8_/S Full Cell

**Figure 5:**
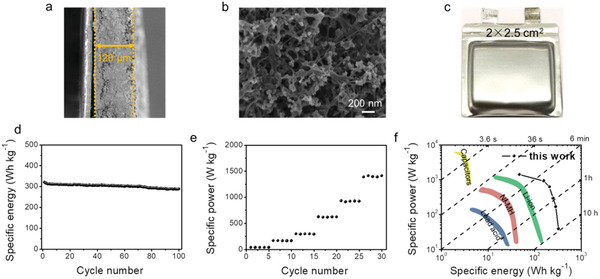
The power and energy densities of the Li@PCMs||N‐Co_9_S_8_/S full cell with high sulfur mass loading. a) Cross‐sectional and b) top‐view SEM images of the N‐Co_9_S_8_/S electrode, c) the photo of Li—S pouch cell, d) the specific energy and e) the specific power of the Li‐S pouch cell, and f) Ragone plot comparing various energy storage devices.

To solve this issue, the last step in our approach is to mix the CNTs with N‐Co_9_S_8_/S cathode. The SEM images of this composite show a relatively large packing density, and the cross‐sectional view shows a thickness of only 120 µm at 6.3 mg cm^−2^ (**Figure** **5a**). The detailed SEM displays that CNTs can support a robust 3D interconnected network (Figure 5b and Figure S12, Supporting Information). Owning to the excellent electrical and mechanical properties, the well‐dispersed CNTs offer an excellent conductive network and also greatly mitigate the volume change during the repeated charging/discharging. Figure 5c shows the photo of a Li@PCMs|N‐Co_9_S_8_/S full pouch cell with a size of 2 × 2.5 cm^2^. And the pouch cell operates at an open‐circuit voltage of 2.468 V (Figure S13, Supporting Information).

The results of high mass loadings are very encouraging. The Li@PCMs||N‐Co_9_S_8_/S full pouch cell demonstrates an extremely high energy density of 325 Wh kg^−1^ when cycled at 0.2 A g^−1^. Even after 100 cycles, the values still maintain at 288 Wh kg^−1^ (Figure 5d). When further increased the current density to 5 A g^−1^, the pouch cell can deliver a relatively high power density of 466 W kg^−1^ (after 100 cycles, Figure S15, Supporting Information). And these results are significantly higher than the previous reports.^[^ ^28^, ^29^ ^]^ It is worth pointing out, that there is still plenty of room for improvement of the energy density and power density, by optimizing the component parameters in a pouch cell.^41,44^ The specific capacity and areal capacity are shown in Figure S14, Supporting Information, which are larger than the traditional C/S based cathode, suggesting an improved sulfur utilization. Even when the sulfur mass loading is increased to 9.8 mg cm^−1^, the specific capacities of both the coin cell and pouch cell still show appreciable values (Figure S16, Supporting Information). This specific capacity represents an extremely high level when compared with the previous Li‐S full cell works (see Table S1, Supporting Information).

The rate performance of the Li@PCMs||N‐Co_9_S_8_/S full cells are also impressive (Figure S17, Supporting Information). The power density of the Li‐S full pouch cell is higher than the previous Li‐S studies (< 1000 W kg^−1^).^[^ ^28^ ^]^ When compared with other main‐stream energy storage technologies, an Li@PCMs||N‐Co_9_S_8_/S pouch cell shows significantly improved performances in terms of the energy and power densities (Figure 5f).^[^ ^4^, ^30^ ^]^ Therefore, the biomass‐based integral approach enables the design of high energy, high power Li‐S batteries for practical applications (e.g., electric vehicles/grid storage).

## 3 Conclusions

In summary, it has been a challenge to realize a practical Li‐S full (pouch) cell with superior energy density and power density. We propose a biomass‐based integral approach to solve this problem. A novel type of biomass‐based PCMs have been fabricated by freeze‐drying and then carbonizing the watermelon flesh, which allows the complete suppression of the Li dendrite growth, and more importantly, a fast Li deposition with large areal capacity. This leads to the significant increase of power density in a Li‐S full cell, without sacrificing the energy density. Combined with the N‐Co_9_S_8_/S cathode that based on the recently developed N‐Co_9_S_8_ nanoparticles and the CNTs, the full cells show unprecedented performances even with high active material mass loadings and under low *E/S* ratios. The state‐of‐the‐art Li@PCMs||N‐Co_9_S_8_/S full pouch cell unlocks the great potentials of both anode and cathode, thus achieving superior energy density and power density (325 Wh kg^−1^ and 1412 W kg^−1^, respectively). This work successfully addresses the well‐known issue of low power densities in Li‐S full (pouch) cells, and therefore bringing the maturity of Li‐S technology to the next level.

## 4 Experimental Section

### Preparation of the Porous Carbon Matrices

A watermelon was cut into small pieces and freeze‐dried over 24 h. Then the freeze‐dried watermelon pieces were carbonized in a tube furnace under the N_2_ atmosphere. The procedure consisted of the first heating to 200 °C at 5 °C min^−1^ and staying at 200 °C for 1 h, and then the second heating to 800 °C at 10 °C min^−1^ and staying at 800 °C for 3 h (to fully carbonize the watermelon). After the full carbonization, the bright‐red watermelon became black pieces. Finally, these black pieces were ground to powder to form the porous carbon matrices.

### Preparation of the Porous Carbon Matrices‐Based Li Anode

The above‐obtained porous carbon matrices were uniformly coated onto the Cu (with 10 wt% PVDF), and then cut into disc‐shaped working electrodes (with a diameter of 12 mm for coin cell, and a size of 2 × 2.5 cm^2^ for pouch cell, the mass loading of PCMs on Cu foil is about 0.2 mg cm^−2^). Li metal melt infusion was carried out in the argon‐filled glovebox under sub‐ppm H_2_O and O_2_ (MBRAUN UNI lab) conditions. First, several polished Li foils were put into a stainless steel crucible and heated on a hotplate at 300 °C. Subsequently, the PCM‐based working electrode was put on top of the molten Li, and the Li melt was infused into the porous structures and the interspace among the porous carbons. The color of the electrode surface changes from black to light yellow, indicating the formation of porous carbon matrices‐Li anode (Li@PCMs). The mass loading of Li can be controlled by the infusion time. Finally, the Li@PCMs anodes were immersed into the electrolyte (1.0 m LiTFSI in DOL/DME with 0.1 m LiNO_3_) for 2 h to form a stable SEI. Furthermore, Li@PCMs anodes can be also prepared by pre‐depositing Li into/onto the PCMs use the half cells. Then the Li@PCMs anodes can be extracted from the half cells, and used in the symmetrical batteries and full cells, separately.

### Preparation of the N‐Co_9_S_8_/S Cathode

The N‐Co_9_S_8_ nanoparticles were prepared according to our recent work.^[^ ^19^ ^]^ Sublimed sulfur was impregnated onto the N‐Co_9_S_8_ nanoparticles by a melt‐diffusion method. The as‐prepared N‐Co_9_S_8_ nanoparticles and sulfur were ground together with a mass ratio of 8:2, and then the mixture was heated at 155 °C for 12 h in a sealed vial to facilitate sulfur diffusion under the argon atmosphere. The carbon black/S (C/S) composite was prepared using the same method as the N‐Co_9_S_8_/S composite. The sulfur electrodes were prepared using a slurry coating method, 80 wt% N‐Co_9_S_8_/S (or C/S), 10 wt% carbon black or CNTs, and 10 wt% PVDF (binder) were mixed in NMP to form a slurry and coated on Al foil and then dried at 60 °C over 12 h. (Note: the low sulfur mass loading electrodes used the carbon black as the conductive additive, while the high sulfur mass loading electrodes used the CNTs as conductive additive. This is because the CNTs can simultaneously boost the electrical and mechanical properties of the thick cathodes.)

### Characterizations

The morphology and microstructure of the samples were investigated by SEM (JEOL JSM‐6700F) and TEM (JEOL JEM‐2100F‐UHR). N_2_ adsorption/desorption isotherms were determined using an ASAP2050 instrument (Micromeritics Instrument Corp). The surface area was determined based on the Barrett–Joyner–Halenda (BET) method. X‐ray diffraction was performed on a PANalytical X‐ray diffractometer at a scanning rate of 0.05° s^−1^. Raman spectroscopy was carried out on the inVia Raman spectrometer from Renishaw with a HeNe laser (632.8 nm excitation wavelength).

### Pouch Cell Assembly

First, both the Li anode and sulfur cathode were cut into pieces with a size of 2 × 2.5 cm^2^. Second, the Ni tab was pressed to attach with the cathode and covered with a separator, and then the Al tab was pressed to attach the anode. Finally, the Li‐S full cell was injected with electrolyte and sealed by Al laminated films.

### Electrochemical Measurements

Both CR2032‐type coin cells and pouch cells were assembled in an argon‐filled glovebox for the electrochemical measurements. 1.0 m lithium bis(trifluoromethanesulfonyl)imide (LiTFSI, Sigma‐Aldrich) dissolved in a mixture 1,3‐dioxolane (DOL, Sigma‐Aldrich) and 1,2‐dimethoxyethane (DME, Sigma‐Aldrich) (1:1 v/v) with 2 wt% LiNO_3_ (Sigma‐Aldrich) additive was used as the electrolyte. The E/S ratio for low sulfur mass loading (about 1 mg cm^−2^) is about 4 µl mg^−2^, for high sulfur mass loading (about 6.3 and 9.8 mg cm^−2^) it is about 1.5 µl mg^−1^.^[^ ^29^ ^]^ Celgard 2400 membrane was used as the separator. The cycling performances of the cells were measured by galvanostatic charge/discharge within the voltage window of 1.7–2.8 V versus Li/Li^+^ (LAND CT 2001A). CV and EIS measurements were conducted by using Metrohm Auto‐lab.

### The Evaluation of Energy/Power Densities

The energy density (*E*_g_) and power density (*P*_g_) can be calculated by using the following equations:

(1) $$E_{g}=\frac{CV}{M}$$

(2) $$P_{g}=\frac{E_{g}}{T}$$

Where *C* is the capacity (mAh), *V* is the average output voltage (V), *M* is the total weight of the cell (g), and *T* is time (h).

## Conflict of Interest

The authors declare no conflict of interest.

## Supporting information

Supporting Information

## Data Availability

Research data are not shared.
