# Supplementary material for: A Biomass‐Based Integral Approach Enables Li‐S Full Pouch Cells with Exceptional Power Density and Energy Density
Source: Adv Sci (Weinh). 2021 May 24;8(14):2101182. doi: 10.1002/advs.202101182 (PMC8292852; doi:10.1002/advs.202101182)
Supplement: Supplementary file 1 — Supporting Information [file ADVS-8-2101182-s001.pdf]

© 2021 Wiley-VCH GmbH

ADVANCED SCIENCE
Open Access

Supporting Information

for *Adv. Sci.*, DOI: 10.1002/advs.202101182

A Biomass-based Integral Approach enables Li-S Full Pouch Cells with Exceptional Power Density and Energy Density

*Yuping Liu, Yvo Barnscheidt, Manhua Peng, Frederik Bettels, Taoran Li, Tao He, Fei Ding, and Lin Zhang**

Supporting Information

A Biomass-based Integral Approach enables Li-S Full Pouch Cells with Exceptional Power Density and Energy Density

*Yuping Liu, Yvo Barnscheidt, Manhua Peng, Frederik Bettels, Taoran Li, Tao He, Fei Ding, and Lin Zhang**

Dr. Y. Liu, Dr. M. Peng, F. Bettels, T. Li, T. He, Prof. F. Ding, Prof. L. Zhang
Institute of Solid State Physics
Leibniz University Hannover
Appelstrasse 2, 30167 Hannover, Germany
*E-mail: l.zhang@fkp.uni-hannover.de

Dr. Y. Liu, Dr. M. Peng, F. Bettels, T. Li, T. He, Prof. F. Ding, Prof. L. Zhang
Laboratory of Nano and Quantum Engineering (LNQE)
Leibniz University Hannover
Schneiderberg 39, 30167 Hannover, Germany

Y. Barnscheidt
Institute of Electronic Materials and Devices
Leibniz University Hannover
Schneiderberg 32, 30167 Hannover, Germany

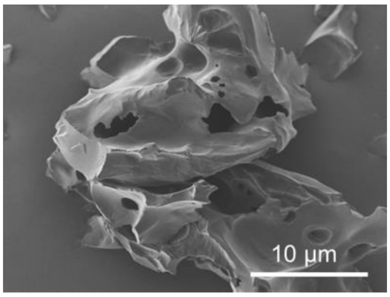

Figure S1. The SEM image of PCM.

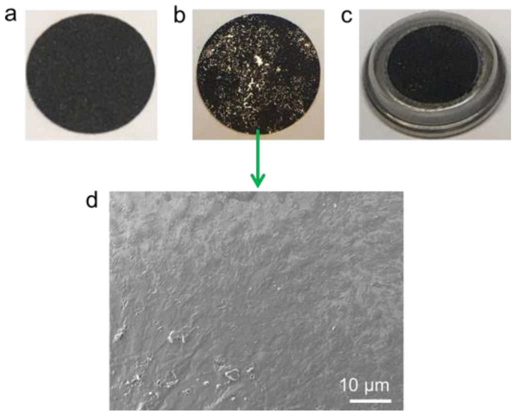

Figure S2. (a) The photo of a piece of PCMs host (coated on the Cu foil), (b) the PCMs host after the Li metal infusion (Li@PCMs), (c) the PCMs host after the Li stripping, (d) the SEM image of the Li@PCMs anode after being immersed into the electrolyte to form stable SEI.

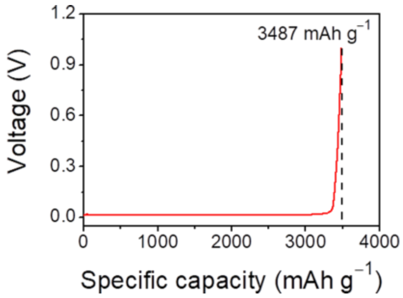

Figure S3. The voltage profile of stripping Li metal for Li@PCMs until 1 V *vs* Li^+/Li.

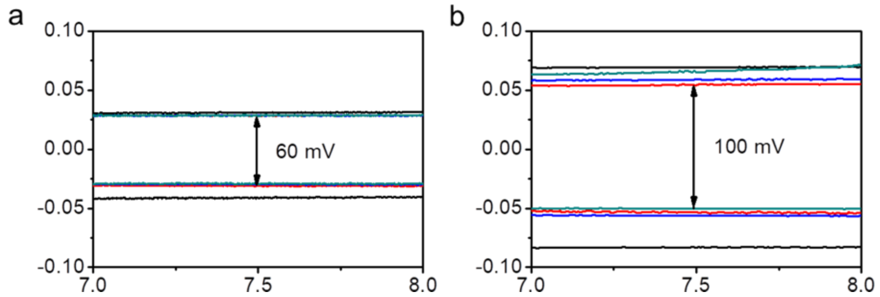

Figure S4. The detailed overpotential of the Li plating/stripping: (a) PCMs and (b) planar Cu.

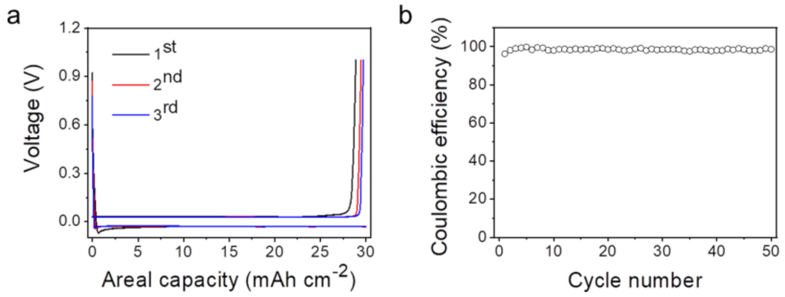

Figure S5. (a) The voltage profiles of the Li plating/stripping for PCMs host and (b) corresponding CE (with an ultra-high areal capacity of 30 mAh cm^{-2} and a current density of 3 mA cm^{-2}).

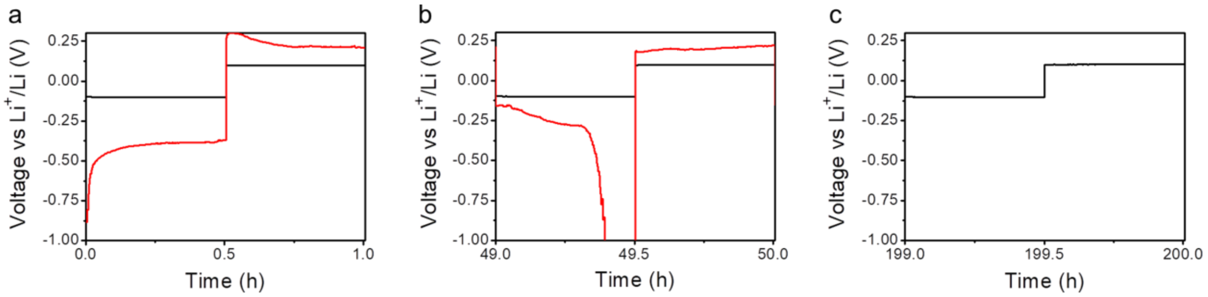

Figure S6. The detailed voltage profiles of the symmetric cell at different cycles in Figure 2h.

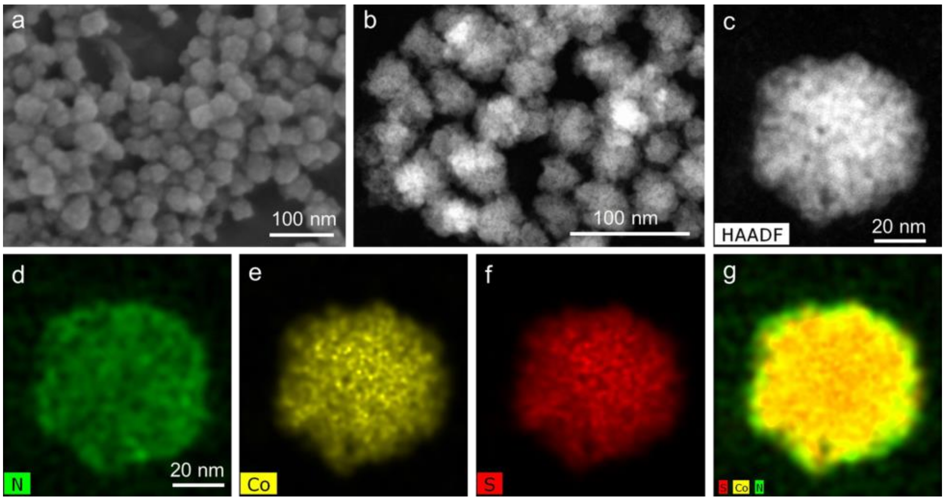

Figure S7. The structural and morphology characterizations of N-Co_9S_8 nanoparticles. (a) SEM, (b) HAADF-STEM, (c) HAADF-STEM for one single N-Co_9S_8 nanoparticle and (d)-(g) elemental mapping images of (d) N, (e) Co, (f) S and (g) their combination for the N-Co_9S_8 nanoparticle.

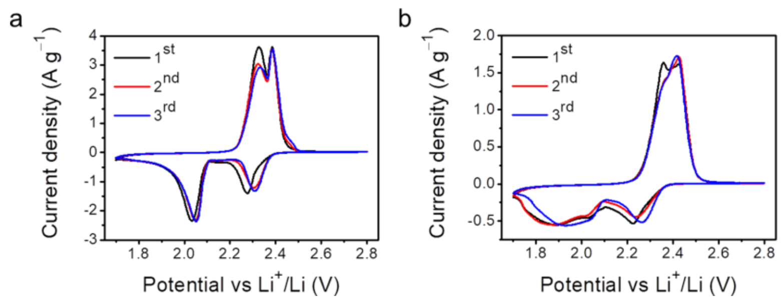

Figure S8. The CV curves of the (a) N-Co_9S_8/S and (b) S electrode (scan rate: 0.1 mV s^{-1}).

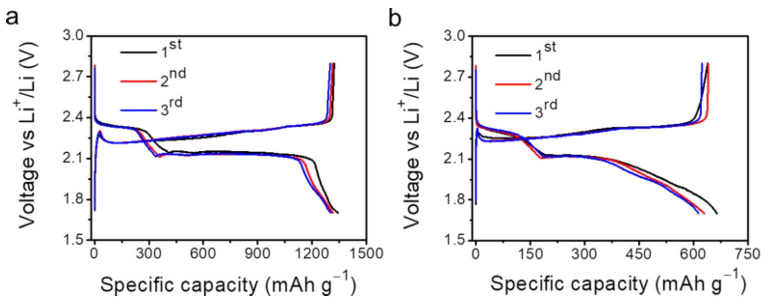

Figure S9. The charge/discharge profiles of the (a) N-Co_9S_8/S and (b) S electrode (current density: 0.2 A g^{-1}).

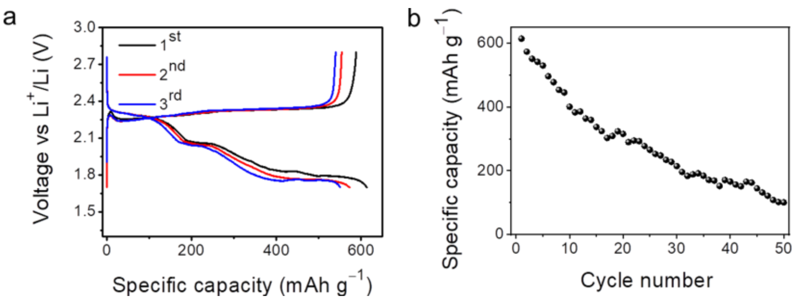

Figure S10. The electrochemical performance of a Li@Cu||S full cell. (a) The charge/discharge profiles and (b) cycling stability (current density: 0.2 A g^{-1}).

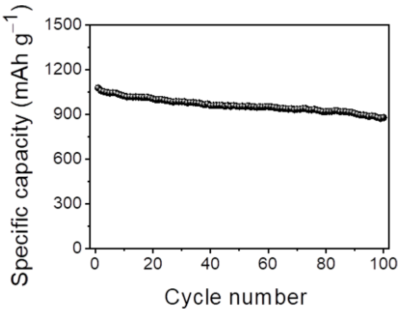

Figure S11. The cycling stability of a Li@PCMs||N-Co_9S_8/S full cell with 6.3 mg cm^{-2} sulfur mass loading (using the carbon black as the conductive additive, current density: 0.2 A g^{-1}).

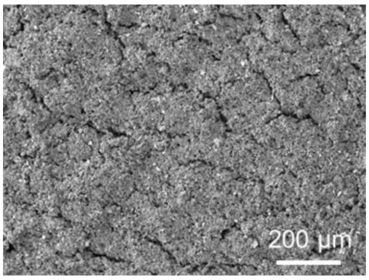

Figure S12. The top-view SEM image of the N-Co_9S_8/S electrode with 6.3 mg cm^{-2} sulfur mass loading.

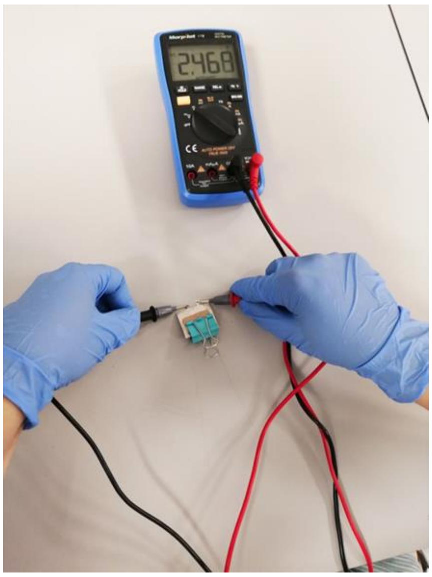

Figure S13. The Li-S pouch full cell shows the open-circuit voltage of 2.468 V.

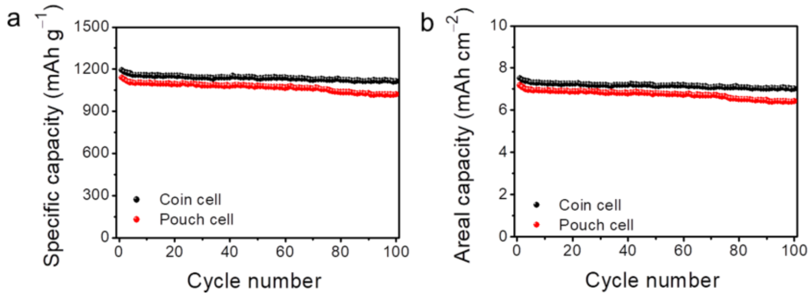

Figure S14. (a) The specific capacity, (b) areal capacity of the Li@PCMs||N-Co_9S_8/S full cell with 6.3 mg cm^{-2} sulfur mass loading (current density: 0.2 A g^{-1}).

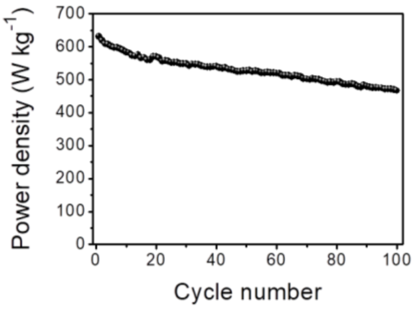

Figure S15. The power density of a Li@PCMs|N-Co_9S_8/S pouch cell with 6.3 mg cm^{-2} sulfur mass loading (current density: 5 A g^{-1}).

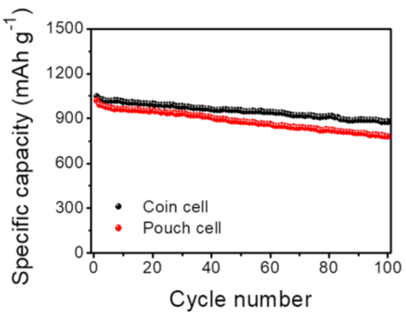

Figure S16. The specific capacity of a Li@PCMs|N-Co_9S_8/S full cell with 9.8 mg cm^{-2} sulfur mass loading (current density: 0.2 A g^{-1}).

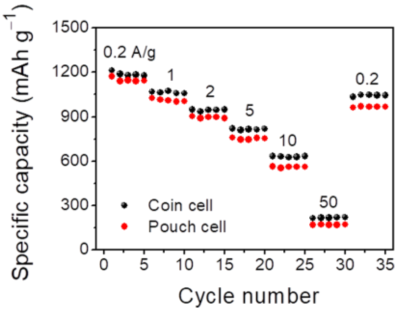

Figure S17. The rate capability of the Li@PCMs||N-Co_9S_8/S full cell with 6.3 mg cm^{-2} sulfur mass loading.

Table S1 Comparison between the Li@PCMs||N-Co_9S_8/S/CNTs full cells with the recently reported Li-S full cells.

Anode	Cathode	Mass loading (mg cm^{-2})	Rate (C)	Capacity retention (mAh/g)	Ref.
Si@VG/Li	S@CB/VGCF	2.0	0.2	775 after 200 cycles	*Nat. Energy* **2016**, *1*, 1.
CF/Ag-Li	S@C	1.7	0.5	650 after 200 cycles	*Energy Environ. Sci.* **2016**, *9*, 3061.
PDMS-Li	S@C	2.5	0.06	761 after 100 cycles	*Nat. Nanotechnol.* **2017**, *12*, 993.
Ti_3C_2-Li	S@C	1.1	0.12	750 after 200 cycles	*Adv. Energy Mater.* **2017**, *7*, 1700260.
Li_xSi/graphene	S@graphitic C	0.7	0.5	850 after 110 cycles	*Adv. Sci.* **2017**, *4*, 1600445.
Li@HPTCF	S@HPTCF	4.0	0.2	775 after 200 cycles	*Adv. Energy Mater.* **2018**, 8, 1802561.
Li/Cu@NPCN	CNT/S	10	0.1	980 after 50 cycles	*ACS Nano* **2019**, 13, 8337.
Li@PCMs	N-Co_9S_8/S/CNTs	6.3<br><br><br>9.8	0.2 A g^{-1}	1114 (coin cell)<br>1018 (pouch cell)<br>after 100 cycles<br>878 (coin cell)<br>779 (pouch cell)<br>after 100 cycles	**This work**
